# Visceral Leishmaniasis with Associated Common, Uncommon, and Atypical Morphological Features on Bone Marrow Aspirate Cytology in Nonendemic Region

**DOI:** 10.1155/2013/861032

**Published:** 2013-09-08

**Authors:** Harish Chandra, Smita Chandra, Rajeev Mohan Kaushik

**Affiliations:** ^1^Department of Pathology, Himalayan Institute of Medical Sciences, Swami Ram Nagar, Doiwala, Dehradun, Uttarakhand 248140, India; ^2^Department of Medicine, Himalayan Institute of Medical Sciences, Swami Ram Nagar, Doiwala, Dehradun, Uttarakhand 248140, India

## Abstract

*Objectives*. The present study was conducted to categorise the morphological features on bone marrow aspirate cytology into common, uncommon, and atypical features in a nonendemic region which would be helpful in clinching an early and correct diagnosis especially in clinically unsuspected cases. *Methods*. The morphological features on bone marrow were categorized into common, uncommon, and atypical in cases of leishmaniasis from non endemic region. *Results*. Out of total 27 cases, 77.7% were residents of places at the height of 500 m or above and fever was the most common presentation followed by hepatosplenomegaly. Plasmacytosis, hemophagocytosis were the common cytological features while dysmyelopoiesis, presence of leishmania bodies in nonhistiocytic cells, and granuloma with necrosis were uncommon features. Aggregates of LD bodies in form of ring, floret, or strap shapes along with giant cells constitute the atypical morphological features. *Conclusion*. The knowledge of common, uncommon, and atypical features on bone marrow aspirate cytology is helpful in clinching an early and correct diagnosis of leishmaniasis especially in non endemic areas where clinical suspicion is low. These features will guide the pathologist for vigilant search of LD bodies in the marrow for definite diagnosis and thus will also be helpful in preventing unnecessary workups.

## 1. Introduction

Visceral leishmaniasis (VL) is vector borne parasitic disease which results from the infection of macrophages in reticuloendothelial system associated with immunoinflammatory response [1]. The higher altitudes negatively affect the distribution of vector, and therefore leishmaniasis is considered to be almost absent in highlands [2]. However, cases demonstrating *Leishmania donovani* (LD) bodies on bone marrow aspirate cytology have been observed in our tertiary care center which caters to nonendemic hilly and subhilly regions. Although bone marrow findings in VL have been described in the literature, studies have rarely categorized these morphological features into common, uncommon, and atypical features according to their frequency and presentation [3–5]. Therefore the present study was conducted to evaluate the clinicohematological profile and study the morphological features on bone marrow examination in cases demonstrating LD bodies in marrow aspirate along with the categorization of these morphological features into common, uncommon, and atypical features. It was also intended to study whether these common, uncommon, and atypical morphological features observed in bone marrow in this nonendemic region differed from prior limited studies that graded the morphological features [3, 4]. This knowledge of morphological categorization would be helpful in clinching an early and correct diagnosis in nonendemic areas especially in clinically unsuspected cases and may prevent the use of advanced and costly diagnostic modalities along with unnecessary workups.

## 2. Material and Methods

The study was conducted in the Pathology department of the institute which included all the cases of leishmaniasis which were diagnosed on bone marrow aspirate cytology by demonstration of LD bodies on Giemsa stained smears over a period of six and half years. Relevant clinical details, investigations, associated morphological features on bone marrow aspirate and trephine biopsy (wherever it was performed), and residential address along with history of visit to endemic regions were recorded for every case. The morphological findings observed on bone marrow aspirate cytology and trephine biopsy were reviewed by two pathologists and were categorized into common, uncommon, and atypical according to the frequency of presentation and as described by prior studies in the literature [3, 4]. The morphological findings were also compared with previous studies especially by Daneshbod et al. which was done in endemic area so as to assess any morphological differences depending on endemicity [4]. Hemophagocytosis (HPS) was graded on aspirate smears as 0-absent; (1+) (mild) <2, histiocytes with HPS/slide; (2+) (moderate) 2–5 histiocytes with HPS/slide; and (3+) (severe) >5 histiocytes with HPS/slide, and average parasite density (APD) was also graded on smears as described in the literature [3].

## 3. Results

The study included total 27 cases of visceral leishmaniasis which were diagnosed on bone marrow aspirate cytology over period of six and half years. Out of these, 22 cases were observed in males and 5 cases in females, and all the cases were of nonendemic region. 18 cases were found in people who were either labourers or farmers or did cattle rearing while 9 cases were employed in various jobs in banks, hospitals, and business or were students. Out of the total 27 cases, 77.7% (21 cases) were residents of places which are at the height of 500 m or above sea level and 8 cases were from 1500 m or above sea level.

Out of the total 27 cases, 22 cases were clinically unsuspected, one case each was associated with human immunodeficiency virus (HIV), tuberculosis, rheumatoid arthritis, and two cases were associated with typhoid.  Table 1 shows the clinical presentation of cases with LD bodies in bone marrow. It shows that fever was the most common presentation followed by hepatosplenomegaly. Tables 2, 3, and 4 show the common, uncommon, and atypical findings observed on peripheral blood smear, bone marrow aspirate cytology, and trephine biopsy examination in cases of leishmaniasis.  Table 5 shows the comparison of hematological and bone marrow findings with previous studies.

## 4. Discussion

**Figure 1:**
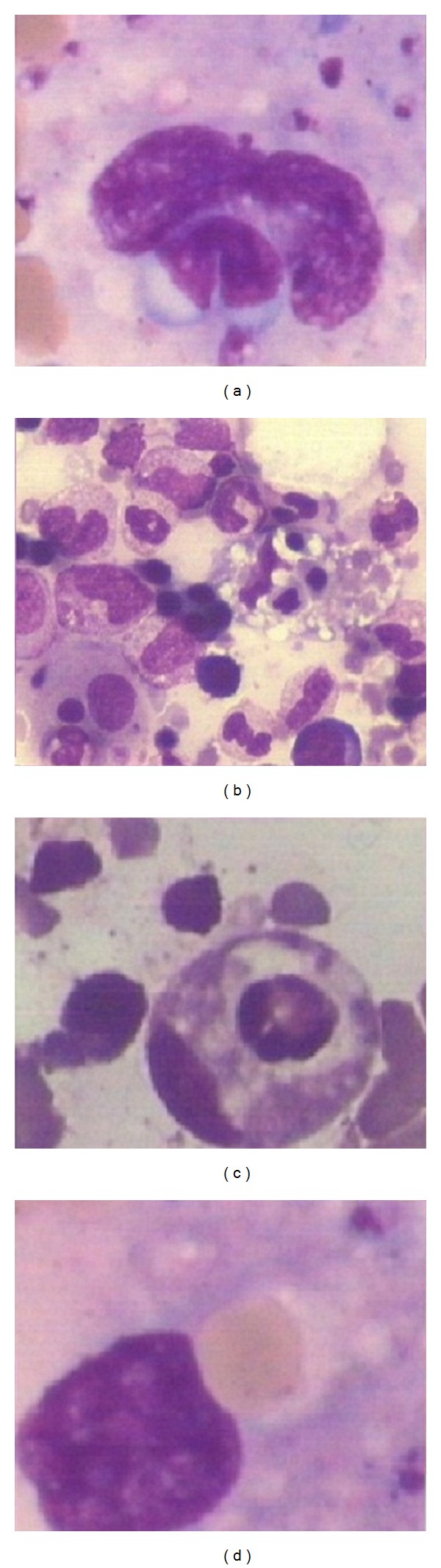
Bone marrow aspirate cytology showing hemophagocytosis in cases of leishmaniasis (Jenner Giemsa; ×400, ×1000).

**Figure 2:**
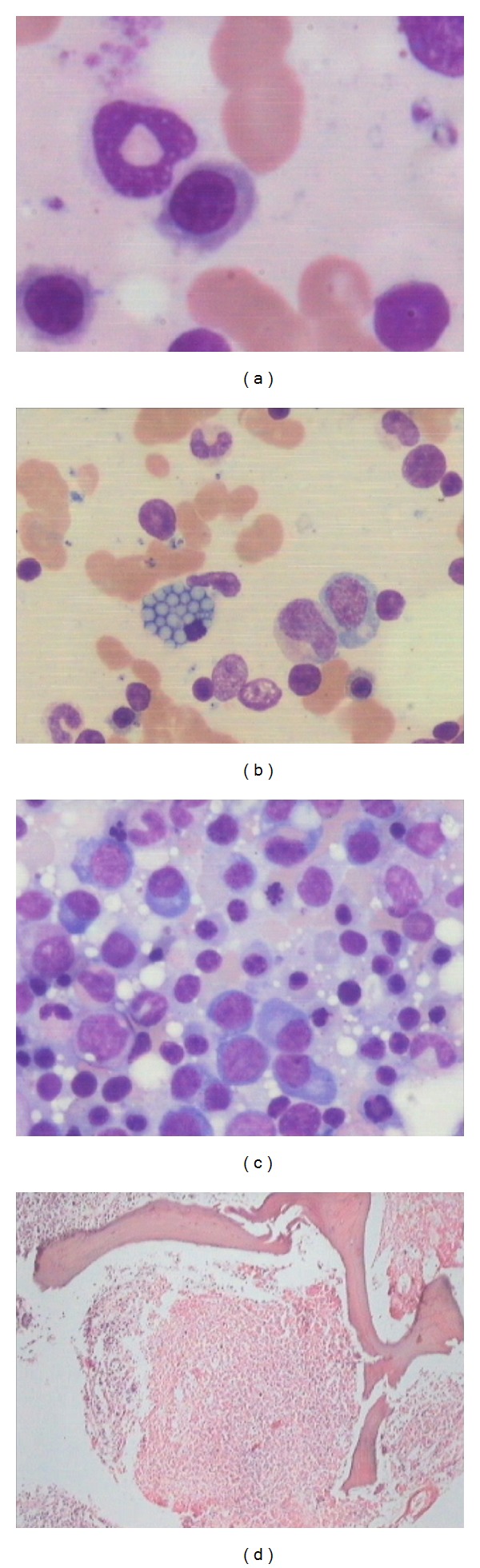
(a) bone marrow aspirate showing dysmyelopoiesis in a case of leishmaniasis, (b) bone marrow aspirate showing plasma cell with grape cell morphology and LD body, (c) bone marrow aspirate showing erythroid hyperplasia with increased plasma cells in leishmaniasis (Jenner Giemsa; ×1000), and (d) bone marrow trephine biopsy showing granuloma and focal necrosis in a case of leishmaniasis (hematoxylin eosin; ×100).

**Figure 3:**
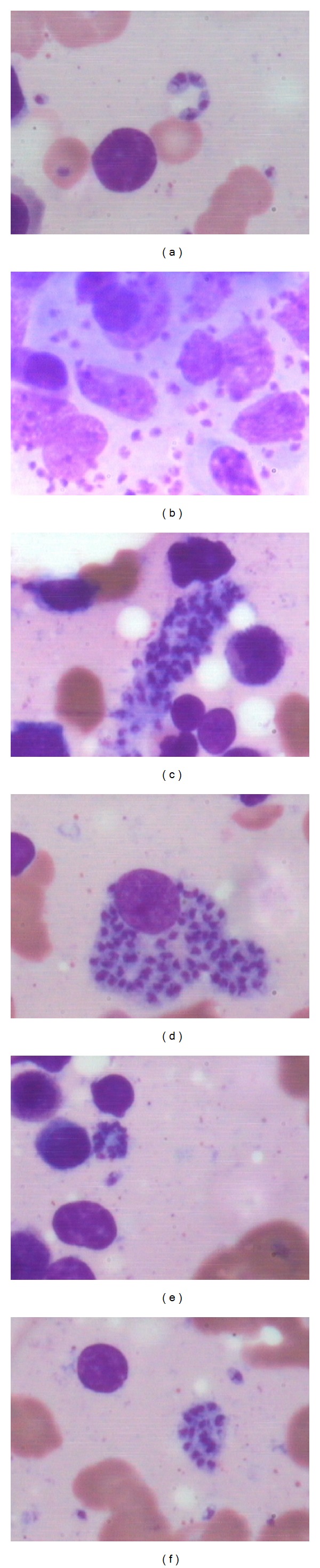
Bone marrow aspirate showing various forms of aggregates of LD bodies, (a) ring shaped aggregate of LD bodies, (b) intracellular and extracellular LD bodies, (c) strap shaped aggregate of LD bodies in cytoplasmic fragment, (d) intracellular aggregates of LD bodies, (e) floret arrangement of LD bodies and (f) LD bodies aggregate in cytoplasmic fragment (Jenner Giemsa; ×1000).

**Figure 4:**
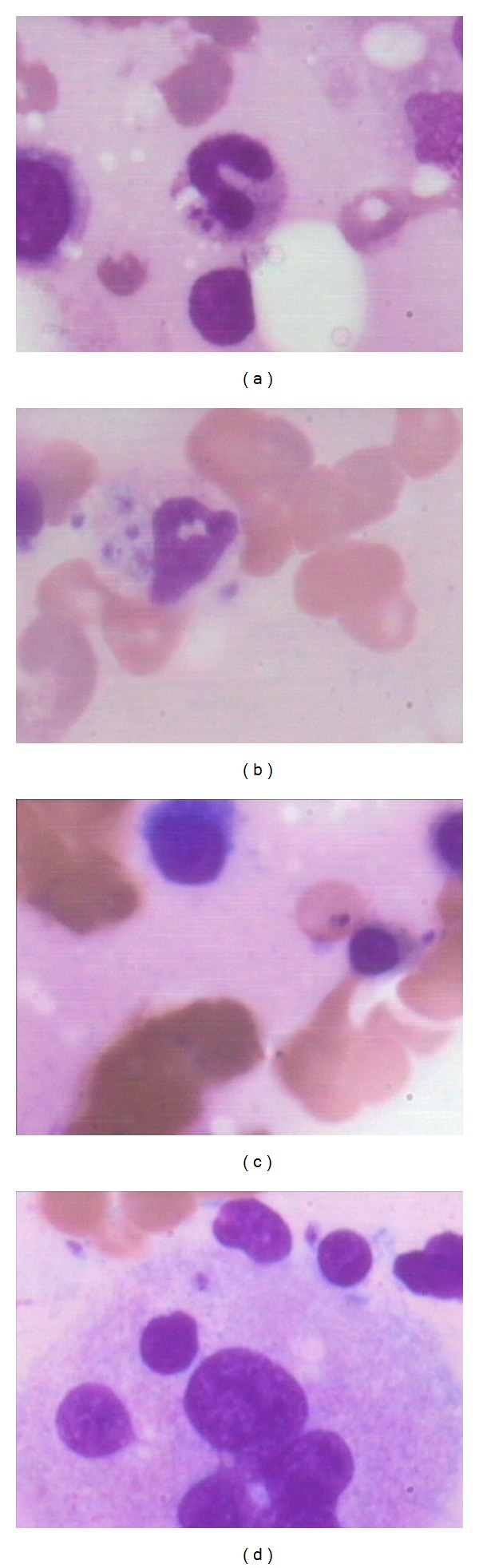
Bone marrow aspirate showing presence of LD bodies in nonhistiocytic cells, (a) LD body in metamyelocyte, (b) LD body in polymorph showing dysmyelopoiesis, (c) LD body in RBC and (d) LD body in megakaryocyte (Jenner Giemsa; ×1000).

Leishmaniasis is considered to be endemic in focal areas in about 90 countries in tropics, subtropics, and Southern Europe while in India, 130 million population is at risk of the disease [1, 6]. Visceral leishmaniasis is considered to be disease of low altitude as climatic and geographical factors play an important role in the distribution of vector, parasite, and reservoir [2, 7]. However, the present study showed that 21 cases were observed from the height of 500 m above sea level, and out of these 8 cases were from regions above 1500 m altitude, thus indicating an emerging focus of disease at higher altitudes. This may either be related to change in environmental factors, population migration, or invasion of the forests. Recently, some studies have also reported new focus of leishmaniasis at higher altitudes of Himalayan and sub-Himalayan regions of India [8–10]. Socioeconomic factors including illiteracy and poverty may be related to the spread of leishmaniasis as 66.6% of cases were present in lower economic group in our study. Fever and hepatosplenomegaly were the common clinical presentation in the present study which is consistent with other studies [11]. However the disease was clinically unsuspected in 81.4% of the cases primarily because its presence in nonendemic areas and secondarily as clinical features overlapped with other prevalent infections of this area such as malaria, enteric fever, and HIV or with liver diseases. This itself lays the importance of vigilant bone marrow aspirate examination for the search of LD bodies and observation of associated bone marrow cytology features even if the clinical suspicion is low. The present study observed that pancytopenia and thrombocytopenia were present in 96.2% cases which is in contrast with another study from endemic region which observed pancytopenia and thrombocytopenia in only 10% and 30% cases, respectively (Table 5) [4]. The probable reason may be delayed diagnosis due to late presentation of cases from remote areas in the study or association with other infections such as tuberculosis and typhoid. On bone marrow aspirate cytology, increased histiocytes and hemophagocytosis were an important common phenomenon observed in the present study (Figure 1) which is in contrast to previous study from Iran by Daneshbod et al. which has reported it to be uncommon cytological findings [4]. However another study has also reported hemophagocytosis as common finding which may be attributed to longer duration of symptoms [3]. Other associated features such as plasmacytosis, erythroid hyperplasia, plasma cells with abnormal inclusions (Russell bodies, Mott cells, and Crystals), and dysmyelopoiesis may also be helpful indicators of leishmaniasis (Figure 2). Aggregates of LD bodies in form of irregular flower shape, ring shape, and strap shape may also be uncommonly observed in leishmaniasis, which at times may mimic fungal spores or platelet aggregates and therefore necessitates a pathologist to have knowledge of such irregular aggregates (Figure 3). Another important difference that was observed from previous study in endemic region by Daneshbod et al. was lesser incidence of free cytoplasmic bodies and granular bodies in the present study (Table 5) [4]. Although the exact cause for this cannot be ascertained, the authors suggest that it may be related to APD which is higher in endemic regions leading to more cytoplasmic disintegration. The release of extracellular cysteine proteinase by amastigotes may be responsible for this cytoplasmic lysis [4]. The present study also indicates that LD body may also be present inside nonhistiocytic cells like polymorph, metamyelocyte, and therefore vigilant search of the parasite in nonhistiocytic cells should also be done (Figure 4). Interestingly, the LD body was also demonstrated in megakaryocyte and red blood cell in our study which has not been reported before (Figure 4). The bone marrow biopsy examination commonly indicates increased vascularity which may be due to reparative process and uncommonly shows necrosis or granuloma (Figure 2) which may be associated due to thrombosis of capillary lumen by parasites [12]. This may be supported by the fact that APD was more in cases showing necrosis on trephine biopsy examination. Daneshbod et al. have observed granuloma (23%) as common feature which is in contrast to the present study which observed granuloma (5.2%) as uncommon feature and it may be attributed to decreased APD in nonendemic region [4]. Although giant cells, necrosis and granulomas are considered to be uncommon and atypical findings in VL in present study, their presence should also prompt close search of LD bodies on bone marrow aspirate cytology (Figure 2). This is especially important in areas were TB is endemic and may be associated with leishmaniasis.

## 5. Conclusion

 Thus to conclude the knowledge of common, uncommon, and atypical features on bone marrow aspirate cytology is helpful in clinching an early and correct diagnosis of leishmaniasis especially in nonendemic areas where clinical suspicion is low. In contrast to previous study from endemic region, hemophagocytosis and pancytopenia were commonly observed while granular and free cytoplasmic bodies were uncommon morphological findings in the present study. The features will guide the pathologist for vigilant search of LD bodies in the marrow aspirate for definite diagnosis. In addition, it will also be useful in preventing the use of advanced and costly diagnostic modalities in the diagnosis of visceral leishmaniasis along with unnecessary workups.

## Figures and Tables

**Table 1:** Clinical presentation in cases showing Leishmania donovani amastigotes in bone marrow.

Clinical presentation	Number of cases	Percentage (%)
Fever	24	88.8
Hepatosplenomegaly	18	66.6
Loss of weight, appetite, and lethargy	08	29.6
Nausea and vomiting	07	25.9
Cough with expectoration	05	18.5
Lymphadenopathy	03	11.1
Diarrhoea	03	11.1
Generalized anasarca	03	11.1
Epistaxis and bleeding per rectum	03	11.1
Miscellaneous (jaundice and joint pains)	03	11.1

**Table 2:** Common hematological and bone marrow findings observed in leishmaniasis.

	Number of cases	Percentage of cases (%)
*Peripheral blood smear *		
Pancytopenia	26	96.2
Anemia	27	100
Leucopenia	26	96.2
Thrombocytopenia	26	96.2
Others (agglutination, fragmented RBC, or Rouleaux formation)	25	92.5
*Bone marrow aspirate cytology *		
Increased histiocytes	27	100
Plasmacytosis	26	96.2
Erythroid hyperplasia	22	81.4
Distribution of LD bodies		
Intrahistiocytic only	00	00
Extrahistiocytic only	01	3.7
Mixed	26	96.2
Hemophagocytosis (mild to moderate)	19	70.3
Average parasite density (1-2+)	24	88.8
*Bone marrow biopsy (total 19 cases) *		
Increased cellularity	05	26
Increased vascularity	06	31.5

**Table 3:** Uncommon hematological and bone marrow findings observed in leishmaniasis.

	Number of cases	Percentage of cases (%)
*Peripheral blood smear *		
Monocytosis	05	18.5
Nucleated RBC	06	22.2
*Bone marrow aspirate cytology *		
Plasma cells with abnormal inclusions	04	14.8
Dysmyelopoiesis	01	3.7
Eosinophilia	04	14.8
Free floating cytoplasm		
With LD bodies	01	3.7
Without LD bodies	04	14.8
Intracellular LD bodies in cells other than histiocytes (polymorph, metamyelocyte, and megakaryocyte)	02	7.4
*Bone marrow biopsy *(*total 19 cases*)		
Necrosis	02	10.5
Granuloma	01	5.2

**Table 4:** Atypical bone marrow findings observed in leishmaniasis.

	Number of cases	Percentage of cases (%)
Bone marrow aspirate cytology		
Aggregates of LD bodies	03	11.1
Kinetoplast only	01	3.7
Giant cells	01	3.7
Bone marrow biopsy (total 19 cases)		
Increased fibrotic foci	02	10.5

**Table 5:** Comparison of hematological and bone marrow findings of the present study with previous studies in leishmaniasis.

	Bhatia et al. [3] (% of cases)	Daneshbod et al. [4] (% of cases)	Present study (% of cases)
*Peripheral blood smear *			
Pancytopenia	75	10	96.2
Anemia	—	70	100
Thrombocytopenia	—	30	100
Leucopenia	—	07	96.2
Monocytosis	62	—	18.5
Nucleated RBC	69	—	22.2
*Bone marrow aspirate cytology *			
Distribution of LD bodies			
Intrahistiocytic	44	—	00
Extrahistiocytic	31	—	3.7
Mixed	25	69	96.2
Plasmacytosis	56	77.9	96.2
Eosinophilia	—	27.4	14.8
Free cytoplasmic bodies			
With LD bodies	—	31.3	3.7
Without LD bodies	—	75.4	14.8
Granular bodies	—	51.9	00
Erythroid hyperplasia	38	78.9	81.4
Increased histiocytes	81	—	100
Hemophagocytosis	75	6.8	70.3
Intracellular nonhistiocytic LD bodies	25	5.9	10.4
Plasma cells with abnormal inclusions	38	2.4	14.8
Increased blasts	06	01	—
Spore like organisms	—	11.8	—
Aggregates of LD bodies	56.2	15.7	11.1
Kinetoplast only	—	8.8	3.7
Pseudo-Pelger-Huet	25	4.9	00
Giant cells	—	7.8	3.7
Atypical histiocytes (Tart cell, foam cells, and RS cells)	25	25.4	00
*Bone marrow biopsy *			
Necrosis	12	2.4	10.4
Granuloma	68	23	5.2
Increased fibrotic foci	19	4.4	10.4
Increased vascularity	63	5.9	31.5
